# Digital Technology Use and BMI: Evidence From a Cross-sectional Analysis of an Adolescent Cohort Study

**DOI:** 10.2196/26485

**Published:** 2021-07-05

**Authors:** Chen Shen, Iroise Dumontheil, Michael Thomas, Martin Röösli, Paul Elliott, Mireille Toledano

**Affiliations:** 1 MRC Centre for Environment and Health Department of Epidemiology and Biostatistics Imperial College London London United Kingdom; 2 National Institute for Health Research Health Protection Research Unit in Chemical and Radiation Threats and Hazards Imperial College London London United Kingdom; 3 Department of Psychological Sciences Birkbeck, University of London London United Kingdom; 4 Department of Epidemiology and Public Health Swiss Tropical and Public Health Institute Basel Switzerland; 5 University of Basel Basel Switzerland; 6 NIHR Biomedical Research Centre Imperial College London London United Kingdom; 7 Mohn Centre for Children’s Health and Wellbeing School of Public Health Imperial College London London United Kingdom

**Keywords:** adolescent, digital technology, obesity, insufficient sleep, mediation analysis, mobile phone

## Abstract

**Background:**

The use of digital technology such as mobile phones is ubiquitous in adolescents. However, excessive use may have adverse health effects, possibly partially mediated by disruptions to sleep.

**Objective:**

This study aims to assess the social predictors of digital technology use and their cross-sectional association with BMI z scores and being overweight in a large sample of adolescents.

**Methods:**

We used baseline data from a subset of a large adolescent cohort from 39 schools across Greater London who participated in the Study of Cognition, Adolescents and Mobile Phones (n=1473). Digital technology use included phone calls, internet use on mobile phones, and video gaming on any device. Multilevel regression was used to assess the associations between digital technology use and age-specific and sex-specific BMI z scores and being overweight (including obesity). Measurements were derived from height and weight, obtained by the Tanita BC-418 Body Composition Analyzer. We examined whether these associations were mediated by insufficient sleep.

**Results:**

Generally, participants with lower socioeconomic status reported more use of digital technology. Controlling for socioeconomic status, internet use on mobile phones for more than 3 hours per day was associated with higher BMI z scores (adjusted β=.30, 95% CI 0.11-0.48) and greater odds of being overweight (adjusted odds ratio 1.60, 95% CI 1.09-2.34), compared with low use (≤30 minutes). Similar associations were found between video gaming and BMI z scores and being overweight. The BMI z score was more strongly related to weekday digital technology use (internet use on mobile phones and video gaming) than weekend use. Insufficient sleep partly mediated the associations between digital technology use and BMI z scores (proportion of mediation from 8.6% to 17.8%) by an indirect effect.

**Conclusions:**

We found an association between digital technology use and BMI in adolescents, partly mediated by insufficient sleep, suggesting that the underlying mechanisms may be multifactorial. Further research with longitudinal data is essential to explore the direction of the relationships.

## Introduction

### Background

With advances in information and communication technologies, mobile phone use is ubiquitous, especially in young people, with 44% of 8- to 11-year olds and 86% of 12- to 15-year olds in the United Kingdom owning a mobile phone [1]. Adolescents aged 12-15 years also spend an average of 12 hours per week on video gaming [1]. Digital technology offers a broad range of functions, facilitating access to the internet and social networks, instant and text message exchange, multimedia, and entertainment. Despite unambiguous advantages such as easy access to information and fast communication, overuse of digital technology is prevalent in children and adolescents because of its powerful potential for diversion and escape from daily life [2,3]. This is becoming a major public health concern with adverse effects on physical health, psychological well-being, and academic achievement in adolescents [4-6].

Despite the exponential increase in digital technology use, the digital divide because of socioeconomic disparity is persistent. However, the nature of the divide today is a gap in the type and intensity of digital technology use, rather than a gap in access to digital devices [7]. Specifically, although family income is not associated with mobile phone ownership in adolescents [8], those with higher family income and parental education are less likely to show problematic mobile phone use or internet addiction as their digital behaviors (eg, time spent on digital devices and digital safety) may be supervised by their parents to a greater extent [9-12]. Adolescents who are marginalized or from disadvantaged groups tend to use digital technology at a higher rate, possibly because they are likely to experience dissatisfaction with their offline lives and web spaces can facilitate the development of relationships with peers with similar life experiences [13,14]. They may also have fewer opportunities to carry out extracurricular activities than their affluent peers and thus spend more time on digital devices [15]. However, there is a paucity of large-scale studies with participants from diverse socioeconomic backgrounds and nuanced exposure measurements to decipher the digital divide regarding various digital technology use, such as phone calls, internet use, and video gaming.

Previous studies have shown that longer duration of phone calls, internet use on mobile phones, and video gaming are associated with a higher risk of overweight and obesity in adolescents [16-19]. However, these studies have small sample sizes [18], do not specify weekday and weekend use [16,17], and only rely on self-reported height and weight, which is not reliable, especially for adolescents whose growth is rapidly changing [16,17,19]. Such limitations preclude a rigorous investigation of the relationship between digital technology use and obesity as well as the underlying mechanism, which is still unclear. The duration of screen-based device use, such as television, mobile phone, and video game consoles, is associated with insufficient sleep duration and increased sleep disturbance in adolescents [20-22]. Possible mechanisms include sleep time displacement by digital technology use and melatonin suppression at bedtime by light exposure emitted from these devices [23]. Insufficient sleep is a well-documented risk factor for obesity in adolescents through the development of insulin resistance, sedentariness, and unhealthy diet in terms of higher consumption of energy-dense foods and sweetened beverages [24]. Therefore, exploration of the complex interrelationships between digital technology use, sleep, and obesity in adolescents is crucial for understanding and preventing obesity.

We have recently established the Study of Cognition, Adolescents and Mobile Phones (SCAMP), the largest adolescent cohort study to investigate the use of mobile phones and other wireless devices and its association with cognitive, behavioral, health, and educational outcomes. We previously observed in this cohort that adolescents who used their mobile phones at nighttime, particularly in darkness, had poorer sleep outcomes compared with nonusers [22].

### Objective

In this study, we aim to examine the associations between digital technology use (eg, phone calls, internet use on mobile phones, and video gaming) and measured BMI outcomes. In addition, we investigated whether insufficient sleep mediated these associations. We hypothesized that increasing digital technology use is associated with higher BMI z scores and being overweight and that the association between digital technology use and BMI z scores is mediated by insufficient sleep.

## Methods

### Study Design and Participants

SCAMP is a prospective adolescent cohort study, with details reported elsewhere [25]. Baseline data were collected from 6616 year 7 pupils (aged 11-12 years), recruited between 2014 and 2016, from 39 secondary schools (26 state and 13 independent) across Greater London, UK. Of all year 7 pupils (N=7375) at these 39 schools, 111 (1.51%) parents or pupils chose to opt out. The remaining nonparticipation (n=648) was because of absence, nonassents by parents, withdrawals, technical issues, or miscellaneous reasons.

Participants in all SCAMP schools completed a computer-based assessment using Psytools software (Delosis Ltd) in examination mode. The assessment included a questionnaire on their digital technology behaviors (eg, smartphone use, social media engagement, and video gaming); a battery of cognitive tests; and health, well-being, and behavior scales. A subset of SCAMP participants (12 out of 39 schools, n=2270) also participated in SCAMP *Bio-Zone* to provide noninvasive biological samples (urine and saliva) and anthropometric measurements (height, weight, waist circumference, grip, and pinch strength) as well as perform a lung function test. The SCAMP *Bio-Zone* participants with measured height and weight (n=1473) were included into the present analysis for assessing the association between digital technology use and BMI. Our sample (n=1473) was powered to detect a small effect (effect size=0.005) for a two-tailed linear regression with 80% power. Our sample size is much larger than the required sample size (n=462) to detect a small mediation effect (eg, β=.14) with 80% power [26].

### Exposure—Digital Technology Use

Categorical responses were provided for questions on digital technology use, for weekdays and weekends separately. Participants who reported using or having used a mobile phone were further asked about the duration of phone calls and internet use (eg, surfing the internet, WhatsApp, Facebook, YouTube, and any other web-based apps) on their mobile phones if their phones were able to connect to the internet. We examined both phone calls and internet use on mobile phones, given the vast majority of mobile phone owners in our study (5119/5490, 93.24%) had a mobile phone that connected to the internet. Internet use on tablets or laptops was not included, as it was not specifically measured. Participants were also asked about the duration of playing video games on any device. We integrated weekday and weekend use to yield average daily use, and categorized internet use on mobile phones and video gaming as 0-30 minutes, 31-59 minutes, 1-2 hours, and 3 hours or more, to allow for a sufficient sample size in each category. Daily use for ≥3 hours was defined as high use [27]. As very few participants reported long duration of phone calls, we categorized responses as 0-5 minutes, 6-15 minutes, 16-59 minutes, and ≥1 hour average daily use.

### Mediator—Insufficient Sleep on Weekdays and Weekends

Adolescents provided information on when they usually got into bed, how long it took them to fall asleep, and what time they usually woke up, separately for weekdays and weekends. Sleep duration was derived from these responses. A sleep duration of less than 9 hours per day was defined as insufficient sleep, based on the recommendation of the US National Sleep Foundation for school-aged children [28].

### Outcome—BMI z Score and Overweight Status at About 12 Years

We measured height (m) and weight (kg) using Tanita BC-418 Body Composition Analyzer. We used age- (in days) and sex-specific BMI z scores based on the 2007 World Health Organization growth reference by interpolating the World Health Organization references on a daily scale [29]. Participants with implausible BMI z scores (SD ≥4 away from the mean) were excluded (n=1). We also defined adolescent overweight and obesity as a BMI for age and sex corresponding to an adult BMI ≥25 and 30 kg/m^2^, respectively, using the International Obesity Task Force cutoffs [30]. Owing to the relatively small number of adolescents with obesity (79/1473, 5.36%), we combined obese and overweight into one category of *overweight* (total n=377).

### Covariates

Demographic information including age, sex, ethnicity (combined into *White*, *Black*, *Asian*, *Mixed*, and *Other*), and parental education and occupation was captured in the SCAMP assessment. Parental education was categorized in a binary form as follows: at least one parent with higher education and no parent with higher education. We used the Office for National Statistics classification of parental occupation, categorizing it into 3 levels [31]. Each child was allocated the higher level of either parent. Dietary factors were also considered as potential confounders. The participants were asked whether they normally eat breakfast. Responses were categorized as *yes* or *no*.

### Statistical Analysis

Ordinal logistic regression yielded adjusted odds ratios (ORs) and 95% CIs for each exposure variable (phone calls, internet use on mobile phones, and video gaming) in relation to demographic characteristics and school type (state or independent). Multilevel linear regression was used to assess the associations between each of the exposure variables and BMI z score, adjusted for potential confounders and school clustering effect. Multilevel logistic regression was used to assess the associations between each exposure variable and overweight.

Potential confounders included age, sex, ethnicity, parental education, parental occupation, and breakfast eating, selected based on directed acyclic graphs (DAGs) [32]. DAG is a simple and transparent way to identify and demonstrate causal relationships between variables via graphs. Confounders were defined as the mutual causes of exposure and outcome variables. Model 1 showed unadjusted associations between each of the exposure variables and BMI z score or overweight. Model 2 was adjusted for age, sex, and ethnicity. In addition, we adjusted for socioeconomic status (SES) indicators (parental education and parental occupation) and breakfast eating in model 3. Missing values for covariates were assigned to a separate *missing* category for each covariate but were not excluded from the analysis. We also examined whether these associations were modified by sex, ethnicity, or parental occupation based on the significance of the interaction terms. We also assessed the associations between weekday and weekend digital technology use and BMI z scores. The *z* test for equality of regression coefficients was performed to assess whether associations with weekday use and weekend use differed.

Causal mediation analysis was performed to examine the role of insufficient sleep as a potential mediator between digital technology use and BMI z scores. Mediation effects of insufficient sleep on weekdays and weekends were analyzed separately. We used VanderWeele formula to compute the direct effect (ie, the effect of digital technology use on BMI z score independent of insufficient sleep), indirect effect (ie, the effect of digital technology use on BMI z score via insufficient sleep), total effect, and the proportion mediated by insufficient sleep [33]. SEs for mediation analysis were estimated using a bootstrap procedure with 5000 bootstrap replications to obtain a 95% CI. All analyses were performed using STATA version IC/13.1 for Windows (Stata Corp).

### Ethical Approval

The North West Haydock Research Ethics Committee approved the SCAMP study protocol and subsequent amendments (reference number 14/NW/0347). Head teachers of schools consented to participation in SCAMP. Parents and adolescents were provided with written information about the study in advance and were given the opportunity to opt out of the research at any time. The study was conducted in accordance with the Declaration of Helsinki.

## Results

### Overview

The median age of our study sample was 12.06 (IQR 11.79-12.33) years. The sample was diverse in terms of ethnicity and SES (Table 1). Almost a fifth of the cohort (1146/6616, 17.32%) spent ≥3 hours per day on internet use and 11.08% (733/6616) on video gaming. A total of 6.71% (444/6616) spent ≥1 hour per day on phone calls. The correlation between measures (eg, phone calls) of digital technology use was low (Cohen <0.3). Of the original 6616 participants in SCAMP, 1473 (22.26%) had measured height and weight. The mean BMI z score in our sample was 0.43 (SD 1.21). We could not find a substantial difference between these participants and the entire cohort in terms of sociodemographic characteristics and digital technology use [25].

**Table 1:** Sociodemographic characteristics, digital technology use (combining weekday and weekend use), and BMI z score of the Study of Cognition, Adolescents, and Mobile Phones cohort.

Sociodemographic characteristics			Overall (N=6616)		Bio-zone participants with measured height and weight (n=1473)
Age (years), median (IQR)			12.06 (11.79-12.33)		12.18 (11.94-12.44)
**Sex, n (%)**					
	Male	3147 (47.57)		747 (50.71)	
	Female	3469 (52.43)		726 (49.29)	
**Ethnicity, n (%)**					
	White	2820 (42.62)		643 (43.65)	
	Black	1016 (15.36)		189 (12.83)	
	Asian	1758 (26.57)		425 (28.85)	
	Mixed	740 (11.19)		160 (10.86)	
	Other	62 (0.94)		17 (1.15)	
	Missing or not interpretable	220 (3.33)		39 (2.65)	
**Parental higher education, n (%)**					
	At least one	3677 (55.58)		891 (60.49)	
	None	1200 (18.14)		265 (17.99)	
	Missing	1739 (26.28)		317 (21.52)	
**Parental occupation, n (%)**					
	Managerial and professional	3426 (51.78)		867 (58.86)	
	Intermediate	1480 (22.37)		300 (20.37)	
	Routine and manual	1040 (15.72)		201 (13.65)	
	Missing	670 (10.13)		105 (7.13)	
**School type, n (%)**					
	State	5141 (77.71)		1035 (70.26)	
	Independent	1475 (22.29)		438 (29.74)	
**Phone calls, n (%)**					
	0-5 min	2236 (33.8)		559 (37.95)	
	6-15 min	1943 (29.37)		405 (27.49)	
	16-59 min	867 (13.1)		190 (12.9)	
	>1 h	444 (6.71)		81 (5.49)	
	Missing	1126 (17.02)		238 (16.16)	
**Internet use on mobile phones, n (%)**					
	0-30 min	2016 (30.47)		459 (31.16)	
	31-59 min	858 (12.97)		183 (12.42)	
	1-2 h	1094 (16.54)		253 (17.18)	
	>3 h	1146 (17.32)		264 (17.92)	
	Missing^a^	1502 (22.7)		314 (21.32)	
**Video gaming on any device, n (%)**					
	0-30 min	3440 (51.99)		788 (53.49)	
	31-59 min	981 (14.83)		233 (15.82)	
	1-2 h	1252 (18.92)		286 (19.42)	
	>3 h	733 (11.08)		124 (8.42)	
	Missing	210 (3.17)		42 (2.85)	
BMI z score, mean (SD)			N/A^b^		0.43 (1.21)

^a^Participants who did not own a mobile phone were recorded as missing data.

^b^N/A: not applicable.

### Associations Between Digital Technology Use and Other Variables

Table 2 shows that after mutual adjustment of all sociodemographic factors, participants at older ages reported longer duration of phone calls and internet use on mobile phones. Girls reported longer duration of phone calls but shorter duration of video gaming compared with boys. Compared with participants of White ethnicity, participants of Black ethnicity reported longer call duration and internet use on mobile phones, but the duration of video gaming was similar between White and Black participants. Asian participants reported lower levels of digital technology use. Participants whose parental occupation was classified as *routine and manual* reported longer duration of phone calls and video gaming than participants with parents in *managerial and professional* occupation. Participants from state schools reported longer duration of digital technology use than those from independent schools.

Table 3 shows that participants who spent ≥1 hour per day on mobile phone calls had 0.42 (95% CI 0.13-0.71) higher BMI z scores than those reporting <5 minutes of use, after adjusting for age, sex, ethnicity, parental education, parental occupation, and breakfast eating (model 3). Tables 3 and 4 show that participants who used the internet on mobile phones for more than 3 hours per day had 0.30 (95% CI 0.11- 0.48) higher BMI z scores and 60% ([1.6-1]/1) higher odds of being overweight (OR 1.6, 95% CI 1.09-2.34) than those reporting <30 minutes of use, respectively. Video gaming for ≥3 hours per day was also associated with higher BMI z scores (β=.26, 95% CI 0.03-0.5) and greater odds of being overweight (OR 1.62, 95% CI 1.03-2.53). The associations between digital technology use and BMI z score and being overweight were not modified by sex, ethnicity, or parental occupation (*P* values for interaction ranged from .08 to .89).

Table 5 shows that internet use on mobile phones and video gaming at high level (ie, more than 3 hours per day) on weekdays were associated with higher BMI z score (β=.30, 95% CI 0-0.61; β=.35, 95% CI 0.07-0.63, respectively). However, associations were not evident between high weekend use and BMI z score. The association between video gaming on weekdays and BMI z score was stronger than that between weekend use and BMI z score (*P*=.009). Stronger association of weekday internet use on mobile phones than weekend use with BMI z score was also observed, although the difference was not significant (*P*=.07).

Table 6 shows that insufficient sleep (both on weekdays and weekends) partly mediated (proportion of mediation ranged from 8.6% to 17.8%) the association between high digital technology use and BMI z score by an indirect effect. High use of digital technology remained associated with BMI z score after adjusting for insufficient sleep, shown as the direct effect.

**Table 2:** Associations between sociodemographic variables and digital technology use (combining weekday and weekend use) in the entire cohort (N=6616)^a^.

Characteristics			Phone calls^b^, aOR^c^ (95% CI)		Internet use on mobile phones^d^, aOR (95% CI)		Video gaming on any device^e^, aOR (95% CI)
**Age**							
	Per year increase	1.16 (1.02-1.32)		1.67 (1.46-1.90)		1.00 (0.88-1.14)	
**Sex**							
	Male	1 (reference)		1 (reference)		1 (reference)	
	Female	1.51 (1.36-1.67)		0.97 (0.88-1.08)		0.17 (0.15-0.19)	
**Ethnicity**							
	White	1 (reference)		1 (reference)		1 (reference)	
	Black	1.70 (1.46-1.97)		1.64 (1.41-1.90)		1.10 (0.95-1.27)	
	Asian	0.65 (0.57-0.74)		0.77 (0.67-0.88)		0.63 (0.56-0.72)	
	Mixed	1.24 (1.05-1.46)		1.24 (1.05-1.47)		1.24 (1.06-1.46)	
	Other	1.64 (0.99-2.73)		1.08 (0.64-1.82)		0.89 (0.55-1.44)	
**Parental higher education**							
	Yes	1 (reference)		1 (reference)		1 (reference)	
	No	0.99 (0.86-1.14)		1.12 (0.97-1.30)		1.04 (0.90-1.19)	
**Parental occupation**							
	Managerial and professional	1 (reference)		1 (reference)		1 (reference)	
	Intermediate	1.09 (0.95-1.24)		1.13 (0.98-1.29)		1.19 (1.04-1.36)	
	Routine and manual	1.19 (1.02-1.39)		1.13 (0.97-1.33)		1.27 (1.09-1.47)	
**School type**							
	Independent	1 (reference)		1 (reference)		1 (reference)	
	State	1.88 (1.65-2.15)		2.7 (2.35-3.11)		2.78 (2.40-3.22)	

^a^Associations adjusted for all other independent variables in the table.

^b^Categorized as 0 to 5 minutes, 6 to 15 minutes, 16 to 59 minutes, and >1 hour.

^c^aOR: adjusted odds ratio, indicating the odds of being in higher categories of each characteristic of digital technology use associated with the independent variables relative to the reference group.

^d^Categorized as 0 to 30 minutes, 31 to 59 minutes, 1 to 2 hours, and >3 hours.

^e^Categorized as 0 to 30 minutes, 31 to 59 minutes, 1 to 2 hours, and >3 hours.

**Table 3:** Associations between digital technology use (combining weekday and weekend use) and BMI z score using multilevel linear regression (n=1473)^a^.

Digital technology use		Model 1^b^, β (95% CI)	Model 2^c^, β (95% CI)	Model 3^d^, β (95% CI)
**Phone calls**				
	0-5 min	0 (reference)	0 (reference)	0 (reference)
	6-15 min	.05 (−0.11 to 0.20)	.06 (−0.10 to 0.21)	.06 (−0.10 to 0.21)
	16-59 min	.04 (−0.16 to 0.24)	.05 (−0.15 to 0.25)	.06 (−0.14 to 0.26)
	>1 h	.40 (0.12 to 0.69)	.42 (0.13 to 0.71)	.42 (0.13 to 0.71)
	*P* value for trend	.03	.03	.03
**Internet use on mobile phones**				
	0-30 min	0 (reference)	0 (reference)	0 (reference)
	31-59 min	.17 (−0.04 to 0.37)	.14 (−0.07 to 0.34)	.15 (−0.06 to 0.35)
	1-2 h	.10 (−0.09 to 0.28)	.07 (−0.11 to 0.26)	.08 (−0.11 to 0.26)
	>3 h	.31 (0.13 to 0.50)	.30 (0.11 to 0.49)	.30 (0.11 to 0.48)
	*P* value for trend	.003	.005	.006
**Video gaming on any device**				
	0-30 min	0 (reference)	0 (reference)	0 (reference)
	31-59 min	.27 (0.10 to 0.45)	.23 (0.05 to 0.41)	.22 (0.04 to 0.40)
	1-2 h	.18 (0.02 to 0.35)	.10 (−0.07 to 0.28)	.10 (−0.07 to 0.28)
	>3 h	.37 (0.14 to 0.60)	.27 (0.03 to 0.50)	.26 (0.03 to 0.50)
	*P* value for trend	<.001	.02	.03

^a^Age- and sex-specific BMI z score, using 2007 World Health Organization growth references for 5-19 years.

^b^Crude model.

^c^Adjusted for age, sex, and ethnicity.

^d^Additionally adjusted for parental education, parental occupation, and breakfast eating.

**Table 4:** Associations between digital technology use (combining weekday and weekend use) and overweight status using multilevel logistic regression (n=1473)^a^.

Digital technology use			Model 1^b^, OR^c^ (95% CI)		Model 2^d^, OR (95% CI)		Model 3^e^, OR (95% CI)
**Phone calls**							
	0-5 min	1 (reference)		1 (reference)		1 (reference)	
	6-15 min	1.03 (0.76-1.40)		1.03 (0.75-1.40)		1.02 (0.74-1.39)	
	16-59 min	1.18 (0.80-1.73)		1.2 (0.81-1.77)		1.23 (0.83-1.83)	
	>1 h	1.46 (0.87-2.45)		1.43 (0.84-2.43)		1.45 (0.85-2.48)	
	*P* value for trend	.15		.17		.15	
**Internet use on mobile phones**							
	0-30 min	1 (reference)		1 (reference)		1 (reference)	
	31-59 min	1.34 (0.89-2.01)		1.33 (0.88-2.00)		1.35 (0.89-2.05)	
	1-2 h	1.22 (0.84-1.77)		1.22 (0.84-1.78)		1.26 (0.86-1.85)	
	>3 h	1.55 (1.08-2.24)		1.57 (1.08-2.28)		1.60 (1.09-2.34)	
	*P* value for trend	.03		.03		.02	
**Video gaming on any device**							
	0-30 min	1 (reference)		1 (reference)		1 (reference)	
	31-59 min	1.42 (1.01-1.98)		1.38 (0.98-1.96)		1.38 (0.98-1.96)	
	1-2 h	1.35 (0.98-1.86)		1.29 (0.91-1.82)		1.29 (0.91-1.83)	
	>3 h	1.68 (1.11-2.56)		1.60 (1.03-2.49)		1.62 (1.03-2.53)	
	*P* value for trend	.005		.03		.03	

^a^Overweight (including obesity) was defined as a BMI for age and sex corresponding to an adult BMI of ≥25 kg/m^2^.

^b^Crude model.

^c^OR: odds ratio.

^d^Adjusted for age, sex, and ethnicity.

^e^Additionally adjusted for parental education, parental occupation, and breakfast eating.

**Table 5:** Associations between weekday and weekend digital technology use and BMI z score (n=1473)^a^.

Digital technology use		Weekday use, β (95% CI)	Weekend use, β (95% CI)
**Phone calls**			
	0-5 min	0 (reference)	0 (reference)
	6-15 min	−.09 (−.29 to .10)	.01 (−.18 to .19)
	16-59 min	−.13 (−.39 to .13)	.28 (.05 to .51)
	>1 h	.13 (−.30 to .56)	.25 (−.11 to .61)
	*P* value for trend	.77	.04
**Internet use on mobile phones**			
	0-30 min	0 (reference)	0 (reference)
	31-59 min	.16 (−.09 to .41)	0 (−.22 to .23)
	1-2 h	.05 (−.23 to .33)	−.08 (−.33 to .18)
	>3 h	.3 (.00 to .61)	−.02 (−.31 to .27)
	*P* value for trend	.05	.73
**Video gaming on any device**			
	0-30 min	0 (reference)	0 (reference)
	31-59 min	.29 (.08 to .49)	−.09 (−.28 to .10)
	1-2 h	.17 (−.08 to .41)	−.10 (−.31 to .12)
	>3 h	.35 (.07 to .63)	−.11 (−.35 to .14)
	*P* value for trend	.01	.40

^a^Adjusted for age, sex, ethnicity, parental education, parental occupation, breakfast eating, and school clustering effect; weekday and weekend use also mutually adjusted.

**Table 6:** Associations between digital technology use (combing weekday and weekend use) and BMI z score mediated by insufficient sleep (n=1473)^a^.

Mediator and exposure			Indirect effect, β (95% CI^b^)	Direct effect, β (95% CI)	Total effect, β (95% CI)	Proportion mediated (%)
**Insufficient sleep on weekdays**						
	**Phone calls**					
		0-5 min	0 (reference)	0 (reference)	0 (reference)	N/A^c^
		6-15 min	.02 (−.01 to .02)	.03 (−.13 to .19)	.05 (−.13 to .19)	N/A
		16-59 min	.03 (.00 to .06)	.10 (−.11 to .32)	.13 (−.08 to .34)	N/A
		>1 h	.05 (.03 to .12)	.48 (.19 to .76)	.53 (.27 to .83)	8.6
	**Internet use on mobile phones**					
		0-30	0 (reference)	0 (reference)	0 (reference)	N/A
		31-59	.02 (.00 to .05)	.12 (−.09 to .33)	.14 (−.07 to .35)	N/A
		1-2 h	.03 (.01 to .06)	.06 (−.13 to .25)	.09 (−.10 to .27)	N/A
		>3 h	.05 (.01 to .10)	.28 (.08 to .48)	.33 (.14 to .53)	15.9
	**Video gaming on any device**					
		0-30 min	0 (reference)	0 (reference)	0 (reference)	N/A
		31-59 min	.01 (−.01 to .03)	.21 (.04 to .39)	.22 (.04 to .40)	N/A
		1-2 h	.02 (.01 to .05)	.12 (−.05 to .31)	.14 (−.03 to .33)	N/A
		>3 h	.05 (.02 to .09)	.26 (−.01 to .53)	.3 (.04 to .57)	15
**Insufficient sleep on weekends**						
	**Phone calls**					
		0-5 min	0 (reference)	0 (reference)	0 (reference)	N/A
		6-15 min	.01 (−.01 to .02)	.04 (−.12 to .20)	.05 (−.12 to .20)	N/A
		16-59 min	.03 (.00 to .05)	.1 (−.10 to .32)	.13 (−.08 to .34)	N/A
		>1 h	.05 (.04 to .13)	.48 (.19 to .76)	.53 (.28 to .83)	9.2
	**Internet use on mobile phones**					
		0-30 min	0 (reference)	0 (reference)	0 (reference)	N/A
		31-59 min	.01 (−.01 to .02)	.12 (−.09 to .33)	.13 (−.09 to .32)	N/A
		1-2 h	.03 (.00 to .06)	.06 (−.13 to .24)	.09 (−.11 to .27)	N/A
		>3 h	.05 (.00 to .09)	.29 (.08 to .48)	.33 (.12 to .52)	14.3
	**Video gaming on any device**					
		0-30 min	0 (reference)	0 (reference)	0 (reference)	N/A
		31-59 min	.01 (−.01 to .03)	.22 (.04 to .39)	.22 (.05 to .41)	N/A
		1-2 h	.02 (.01 to .07)	.13 (−.05 to .31)	.15 (−.02 to .34)	N/A
		>3 h	.05 (.03 to .12)	.25 (−.03 to .52)	.31 (.04 to .58)	17.8

^a^Adjusted for age, sex, ethnicity, parent education, parent occupation, breakfast eating, and school type.

^b^CI was obtained by 5000 bootstrap resamples.

^c^N/A: not applicable.

## Discussion

### Principal Findings

Our results showed that Black participants, those with lower SES in terms of parental occupation, and those from state schools use digital technology to a greater extent than White participants, those with higher SES, and those from independent schools, respectively. This study also found that high use of digital technology, including internet use on mobile phones and video gaming, was associated with higher BMI z scores and greater odds of being overweight in adolescents, using objective height and weight measurements. The associations were consistent across sex, ethnicity, and SES. The BMI z score was more strongly related to high weekday digital technology use than high weekend use. Insufficient sleep on weekdays and weekends partly mediated the associations between high digital technology use and BMI z scores.

In line with previous research [9,10], this study found that adolescents whose parents had management and professional occupations used digital technology for a shorter duration. These parents may be more aware of the adverse health consequences of excessive digital engagement and potentially exert more control on and guidance to their children to avoid overuse. Moreover, they may be equipped with advanced digital skills to manage their children’s technology activities. Parents with lower SES, who can be under greater economic and time pressures, likely rate the restriction of children’s screen time and support of children’s web-based activities as relatively low priority compared with other life pressures [34]. Compared with White adolescents, Black adolescents used mobile phones for a longer duration. Black adolescents may have a sense of being marginalized in a school with primarily White students (as in our sample) [35]. The use of mobile phones to connect with family and friends is convenient to broaden and intensify their social support networks. This argument might also explain the different association patterns between ethnicity and video gaming, as the role of video gaming in social support is less pronounced than mobile phone use in general. Participants in state schools used digital technology more than those in independent schools, which is consistent with another study on British adolescents [3]. Although the state schools in our study generally have more restrictions on digital technology use than independent schools (ie, students are strictly not allowed to use their mobile phones during school time), participants from state schools might use digital technology more during weekday evenings.

Screen time, such as television watching, mobile phone use, and video gaming, has been associated with adolescent obesity [16,17,36]. However, inferences from these studies are limited because of the bias of self-reported weight and height as well as a lack of differentiation between weekday and weekend use. To our knowledge, this is the first study to distinguish between weekday and weekend use and investigate a plausible mechanism (insufficient sleep) to help explain the association between digital technology use and adolescent obesity.

This study adds to the literature by showing that weekday use of digital technology might play a more important role than weekend use in individual differences in BMI z scores. Previous research has found that biomarkers related to obesity (eg, insulin and Homeostatic Model Assessment for Insulin Resistance) are more strongly associated with weekday digital technology use than weekend use in adolescents [37]. This is possibly because of the metabolic risks attributed to prolonged sitting bouts already required during class time on weekdays [38]. This study also adds to the literature by showing that insufficient sleep (both on weekdays and weekends) partly explained the associations between digital technology use and BMI z scores through an indirect effect. Plausible explanations include that excessive use of digital technology may displace sleep time, particularly on weekdays. This is because most schools restrict the use of mobile phones during school time, and participants need to wake up early to attend school; therefore, the main time during which participants can use their phones on weekdays is during the evenings and nights. Although direct sleep time displacement is less likely on weekend nights, sleep loss might also be because of psychological and physiological arousal by the content as well as melatonin suppression caused by screen light [21,23]. Notably, the association between digital technology use and BMI z score was still evident after adjusting for insufficient sleep (ie, direct effect), indicating that the underlying mechanisms may extend beyond sleep deprivation. High engagement in digital technology may also displace physical activity, resulting in obesity [39]. Mobile phone and internet addiction, and video game dependency, may act as a manifestation of psychosocial stress, as digital technology may provide a means for adolescents to be distracted from stressful experiences [40-42]. The stress-induced hypothalamic-pituitary-adrenal axis could stimulate appetite and disrupt metabolism and thus promote weight gain [43,44].

This study has several strengths and weaknesses. It is based on the largest prospective cohort of adolescents worldwide, with detailed data on mobile phone technologies. The cohort is representative of school-aged children across Greater London. Analyses used objective measurements for height and weight and did not rely on self-reported information. The study was able to distinguish between weekday and weekend use and investigate a plausible mechanism of insufficient sleep to aid understanding of the association between digital technology use and adolescent obesity. However, this study is subject to residual confounding because of the nature of the observational study design, although confounders were rigorously selected on a scientific basis (ie, based on the DAG). In addition, information on physical activity, other dietary factors (eg, snacking), and psychosocial stressors was not available; therefore, other potential mechanisms could not be considered. We analyzed cross-sectional baseline data of the SCAMP sample; thus, we were not able to detect the temporal sequence of digital technology use and obesity. Longitudinal studies have shown that greater screen time is associated with increased changes in BMI and a higher risk of obesity in adolescents [19,45]. However, we cannot exclude the possibility of obesity leading to sedentary behaviors, such as high engagement in digital technology [46]. Baseline data were collected a few years ago and may, therefore, not reflect the contemporary digital environment, which is rapidly changing. However, we expect our findings to be relevant as screen time has been consistently associated with adolescent obesity in the literature [16,19,47,48]. The age range of our participants was small; therefore, our findings may not be generalizable to older adolescents. Finally, information on mobile phone use, video gaming, and sleep was derived from self-reports, possibly resulting in social desirability bias and measurement error.

This study suggests several avenues for future research. We plan to analyze longitudinal SCAMP data to investigate the temporal sequence of digital technology use and obesity and compare the relationship between digital technology use and obesity at different ages to create age-specific policy recommendations regarding digital technology activities. In addition, more comprehensive measurements of other obesity-related lifestyle factors such as physical activity and psychosocial stressors are important to unravel potential biological and behavioral mechanisms linking digital technology use and adolescent obesity. Objective measures of screen time and a secondary measure of sleep collected using a wearable fitness tracker (eg, Fitbit monitor), in addition to self-reported information, are needed to minimize self-report bias.

Our findings also have significant public health implications. The evidence of the potential impacts of digital technology overuse on adolescent obesity via insufficient sleep will allow the development of guidance for children and adolescents’ healthy use of digital technology to prevent obesity. It may be possible to maintain the level of digital technology use but mitigate its effects on health by altering patterns of use. In addition, the recent COVID-19 pandemic led to school closure, lockdown, and social distancing, which may substantially increase screen time and decrease opportunities for social and physical activity during this period [49]. Schools, teachers, and parents should be vigilant of the secondary impacts of COVID-19 on risks of sleep disturbance and obesity and better advise their students and children on digital technology use.

### Conclusions

This study shows the socioeconomic disparities of digital technology use among 11-to 12-year olds. High use of digital technology, that is, >3 hours per day, was associated with higher BMI z scores and greater odds of being overweight. In general, BMI z scores were more strongly related to high weekday digital technology use than high weekend use. The association between digital technology use and BMI z score was partly mediated by insufficient sleep, suggesting that the underlying mechanisms are multifactorial. Further research on longitudinal data is essential to explore the direction of relationships. Schools, parents, public health practitioners, policy makers, and adolescents themselves should be aware of the potentially adverse health effects of excessive digital technology engagement on sleep and obesity.

## Abbreviations

DAG: directed acyclic graph
NIHR: National Institute for Health Research
OR: odds ratio
PRP: Policy Research Program
SCAMP: Study of Cognition, Adolescents and Mobile Phones
SES: socioeconomic status
